# Engineering Salidroside Biosynthetic Pathway in Hairy Root Cultures of *Rhodiola crenulata* Based on Metabolic Characterization of Tyrosine Decarboxylase

**DOI:** 10.1371/journal.pone.0075459

**Published:** 2013-10-04

**Authors:** Xiaozhong Lan, Kai Chang, Lingjiang Zeng, Xiaoqiang Liu, Fei Qiu, Weilie Zheng, Hong Quan, Zhihua Liao, Min Chen, Wenlin Huang, Wanhong Liu, Qiang Wang

**Affiliations:** 1 Agricultural and Animal Husbandry College, Tibet University, Nyingchi of Tibet, P. R. China; 2 Key Laboratory of Eco-environments in Three Gorges Reservoir Region (Ministry of Education), Chongqing Engineering and Technology Research Center for Sweetpotato, School of Life Sciences, Southwest University, Chongqing, P. R. China; 3 School of Pharmaceutical Sciences, Southwest University, Chongqing, P. R. China; 4 School of Chemistry and Chemical Engineering, Chongqing University of Science and Technology, Chongqing, P. R. China; 5 China Rural Technology Development Center, Ministry of Science and Technology, Beijing, P. R. China; The University of Kansas Medical center, United States of America

## Abstract

Tyrosine decarboxylase initializes salidroside biosynthesis. Metabolic characterization of tyrosine decarboxylase gene from *Rhodiola crenulata* (*RcTYDC*) revealed that it played an important role in salidroside biosynthesis. Recombinant 53 kDa RcTYDC converted tyrosine into tyramine. *RcTYDC* gene expression was induced coordinately with the expression of *RcUDPGT* (the last gene involved in salidroside biosynthesis) in SA/MeJA treatment; the expression of *RcTYDC* and *RcUDPGT* was dramatically upregulated by SA, respectively 49 folds and 36 folds compared with control. MeJA also significantly increased the expression of *RcTYDC* and *RcUDPGT* in hairy root cultures. The tissue profile of *RcTYDC* and *RcUDPGT* was highly similar: highest expression levels found in stems, higher expression levels in leaves than in flowers and roots. The gene expressing levels were consistent with the salidroside accumulation levels. This strongly suggested that RcTYDC played an important role in salidroside biosynthesis in *R*. *crenulata*. Finally, *RcTYDC* was used to engineering salidroside biosynthetic pathway in *R*. *crenulata* hairy roots via metabolic engineering strategy of overexpression. All the transgenic lines showed much higher expression levels of RcTYDC than non-transgenic one. The transgenic lines produced tyramine, tyrosol and salidroside at higher levels, which were respectively 3.21–6.84, 1.50–2.19 and 1.27–3.47 folds compared with the corresponding compound in non-transgenic lines. In conclusion, *RcTYDC* overexpression promoted tyramine biosynthesis that facilitated more metabolic flux flowing toward the downstream pathway and as a result, the intermediate tyrosol was accumulated more that led to the increased production of the end-product salidroside.

## Introduction

*Rhodiola crenulata* is a perennial herbaceous plant and mainly grows on the very high mountains of Tibet Plateau. The stems of *R*. *crenulata* plants have been used as health food and herb more than 1000 years before by Chinese people especially the local Tibetan peoples because it is considered to be the best one of *Rhodiola* species [1]. *R*. *crenulata* belongs to the family of *Crassulaceae* and mostly grows in the very harsh environments with high altitude of 5000 meters and above, low oxygen concentration, strong ultraviolet radiation and poor soils [2]. So, *R*. *crenulata* grows very slowly and can not be found easily in the wild. With more phytochemical and pharmaceutical discoveries in *R*. *crenulata*, it is considered to be a plant-derived adaptogen that is capable of maintaining physiological homeostasis upon exposure to stress [3]. Now *R*. *crenulata* is widely used as anti-depressive, and anti-fatigue and to reinforce immunity, improve memory and learning, scavenge active-oxygen species, and relieve altitude sickness because of its pharmaceutical natural products, salidroside [4] and tyrosol [5]. Due to the limited plant resource of *R*. *crenulata* and the huge commercial demands for *R*. *crenulata*, this magic plant species is endangered. It is absolutely necessary and urgent to find some other alternative ways to produce salidroside. Metabolic engineering salidroside biosynthetic pathway might be the best method.

**Figure 1:**
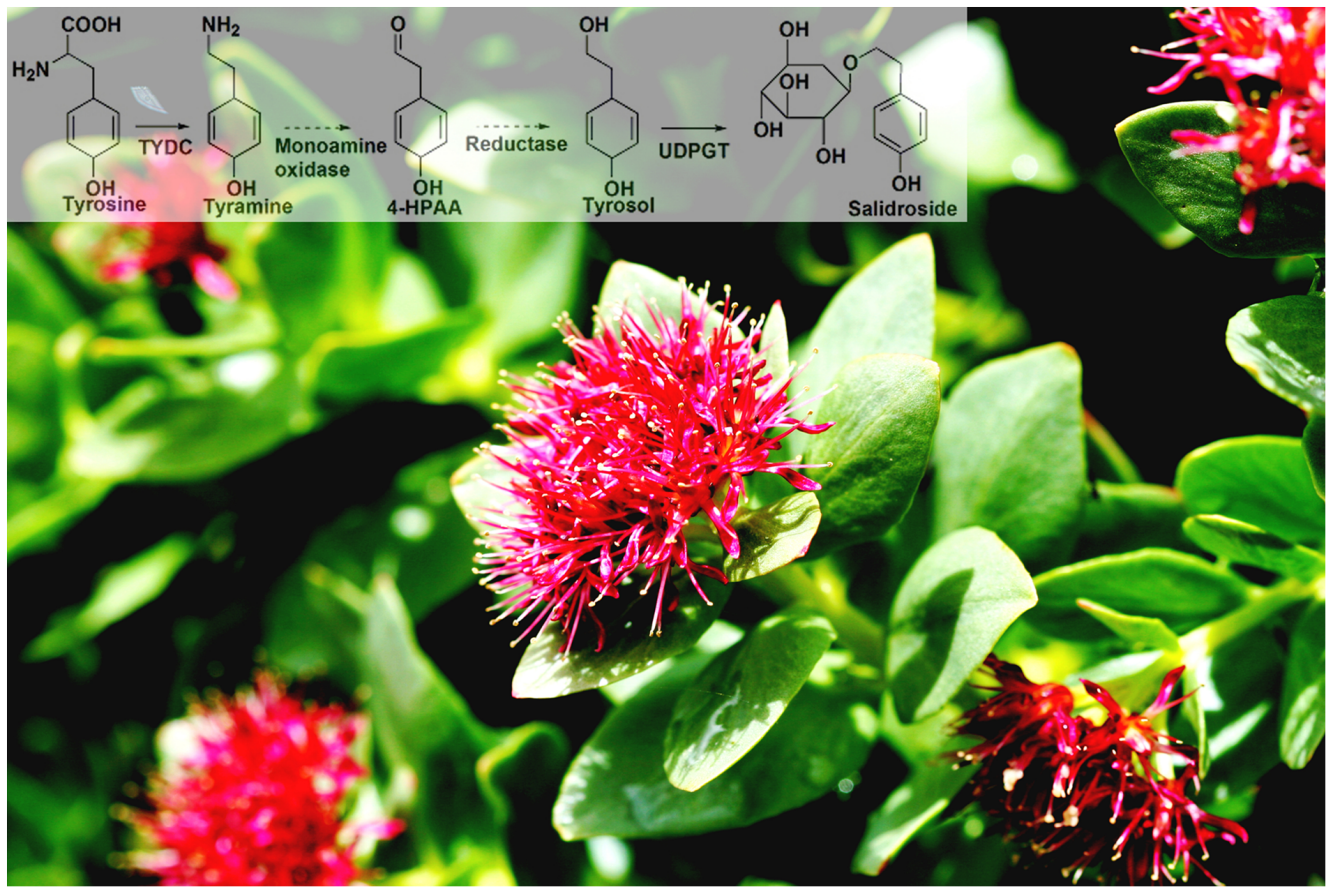
The putative biosynthetic pathway of salidroside and *Rhodiola crenulata*. The arrows indicated the cloned genes and the dashed arrows showed the unidentified genes; the background photo was flowering *Rhodiola crenulata*. TYDC: tyrosine decarboxylase; UDPGT: UDP-glucosyltransferase.

Tyrosine is one of the precursors of salidroside in *Rhodiola* species, which is converted into tyramine by tyrosine decarboxylase (TYDC). Then, tyramine can be converted into tyrosol after two-step enzymatic reactions, which are respectively catalyzed by tyramine oxydase and ary alcohol dehydrogenase. Finally, tyrosol becomes salidroside (8-*O*-*β*-D-glycoside of tyrosol) after glucosylation catalyzed by UDP-glycosyltransferase (Figure 1). TYDC belongs to the family of aromatic L-amino acid decarboxylases and operates at an interface between primary and secondary metabolism, suggesting that it possesses key regulatory functions in the control of end-product biosynthesis [6]. A putative TYDC gene was isolated from Rhodiola rosea, of which expression level was consistent with accumulation of salidroside [7]. TYDC as a key role in tyramine-derived compounds was supported by the study of overexpression of parsley TYDC in potato leading to higher tyramine-derived compounds in potato [8]. In the present study, a full-length cDNA encoding TYDC was cloned and functionally identified by feeding intermediate to recombinant protein from Tibet-specific *R. crenulata*; gene expression profiles were analyzed in different tissues and in hairy root cultures with the treatment of different elicitors; the accumulation patter of salidroside was also investigated; and finally based on the discoveries above, metabolic engineering strategy of overexpressing *RcTYDC* was used to engineering salidroside biosynthetic pathway in hairy root cultures of *R. crenulata* that definitively improved biosynthesis of tyramine, tyrosol and the end-product salidroside.

## Results

### Cloning of the Full-length cDNA of RcTYDC

**Figure 2:**
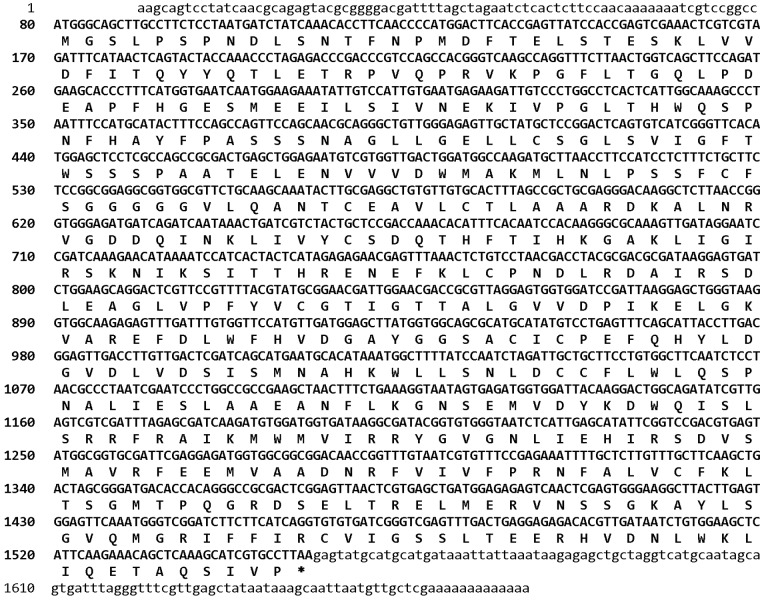
The full-length cDNA of RcTYDC and its deduced amino acids. The coding sequence of RcTYDC was shown with capital letters in bold fonts; the untranslated regions were in small letters; and the stop codon was marked with an asterisk.

A 678-bp cDNA fragment was amplified with a pair of degenerate primers, dfp and drp. The sequence analysis showed that the 678-bp cDNA fragment belonged to the aromatic amino acid decarboxylase superfamily, in which tyrosine decarboxylase was included. The deduced amino acid sequence of the 678-bp cDNA fragment was respectively similar with the amino acid sequence of tyrosine decarboxylase from *Populus trichocarpa* (60% identity), *Vitis vinifera* (55% identity) and *Citrus* species (51% identity). This suggested that the core fragment of *RcTYDC* was obtained that could be used to isolate the full-length cDNA by RACE technology. With the nested 5′ RACE, a 516-bp 5′ cDNA end was specially amplified; and with the nested 3′ RACE, a 533-bp 3′ cDNA end with a poly A tail was isolated. The three cDNA fragments were assembled to generate a 1670-bp full-length cDNA of RcTYDC that was physically confirmed by a pair of primers, fRcTYDC and rRcTYDC. The full-length RcTYDC cDNA had a 1473-bp coding sequence that encoded a 490-amino-acid polypeptide (Figure 2). The 5′ untranslated region was 79 bp, and the 3′ untranslated region was 118 bp with a 13-bp polyA tail (Figure 2). The full-length cDNA of *RcTYDC* was submitted to GenBank and assigned the accession number AFN89854.1.

### Sequence Analysis of RcTYDC

**Figure 3:**
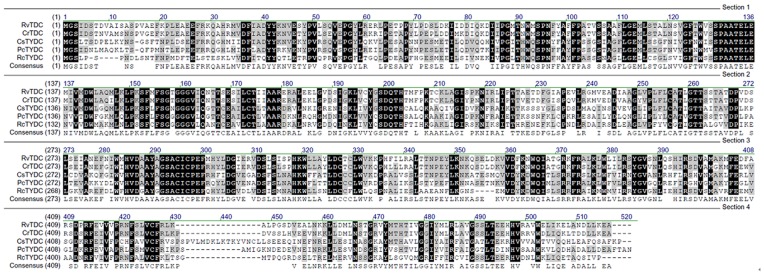
Sequence comparison of plant TYDCs and TDCs. RvTDC: *Rauvolfia verticillata* tryptophan decarboxylase; CrTDC: *Catharanthus roseus* tryptophan decarboxylase; PcTYDC: *Petroselinum crispum* tyrosine decarboxylase; RcTYDC: *Rhodiola crenulata* tyrosine decarboxylase.

**Figure 4:**
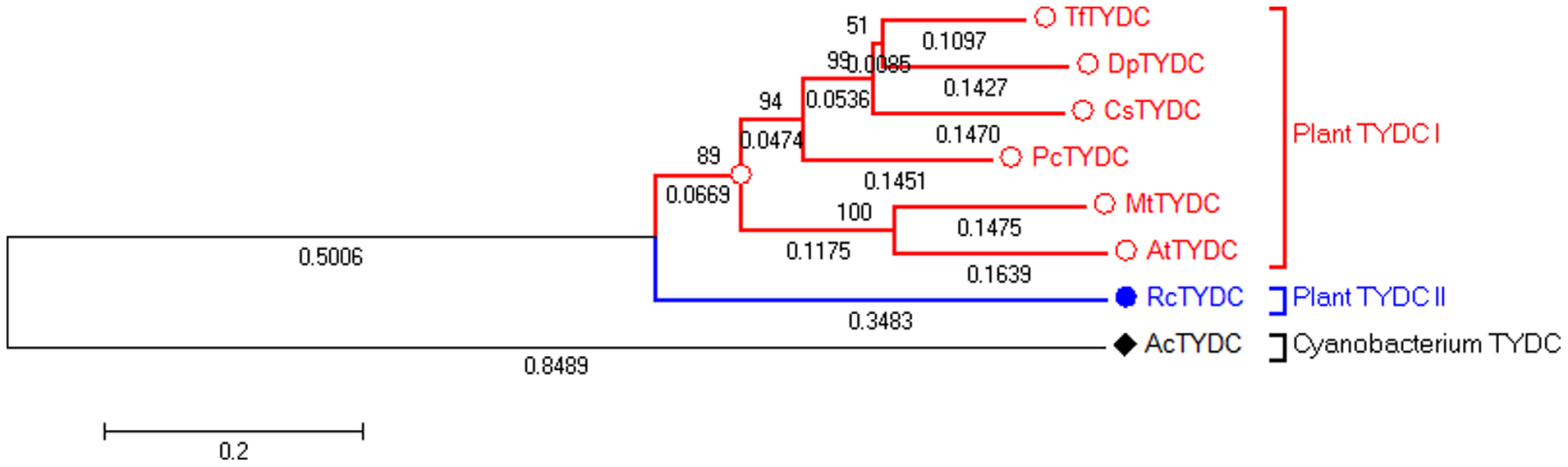
The phylogentic analylsis of plant and cyanobacterium TYDCs. AcTYDC: *Anabaena cylindrical* TYDC (AFZ60767); AtTYDC: *Arabidopsis thaliana* TYDC (CAB56038); CrTYDC: *Citrus reticulata* TYDC (ACX29991); MtTYDC: *Medicago truncatula* TYDC (XP_003625397); PcTYDC: *Petroselinum crispum* TYDC (Q06086); PsTYDC: *Papaver somniferum* TYDC (AAC61843); RcTYDC: *Rhodiola crenulata* TYDC (AFN89854.1); TfTYDC: *Thalictrum flavum* TYDC (AAG60665); VvTYDC: *Vitis vinifera* TYDC (CAN61896).

The full-length cDNA of *RcTYDC* encoded a 490 amino-acid polypeptide with the calculated molecular weight of 53 kDa and theoretical pI of 5.55. The amino acid sequence of RcTYDC was similar (nearly 50% identity) with that of reported TYDCs from other plants such as *Citrus* species [9] and *Opium poppy* [10]; and RcTYDC also showed similarity with tryptophan decarboxylases from *Catharanthus roseus* (48.2% identity) and *Rauvolfia verticillata* (46.3% identity). Therefore, it was impossible to confirm that the gene of *RcTYDC* encoded the enzyme of TYDC only by sequence comparison analysis (Figure 3) and necessary to identify its function by feeding intermediate to the recombinant RcTYDC. A phylogenetic tree was constructed according to the TYDCs from plants and cyanobacterium (Figure 4). The plant TYDCs were separated from cyanobacterium TYDC. The plant TYDCs could be divided into two groups. RcTYDC was separated from all the other plant TYDCs and all the other plant TYDCs were grouped together. This suggested that there might be two types of TYDCs in plants. RcTYDC was a new type of TYDC (plant TYDC II) that was genetically different from the other plant TYDCs (plant TYDC I).

### Identification of the Recombinant RcTYDC

**Figure 5:**
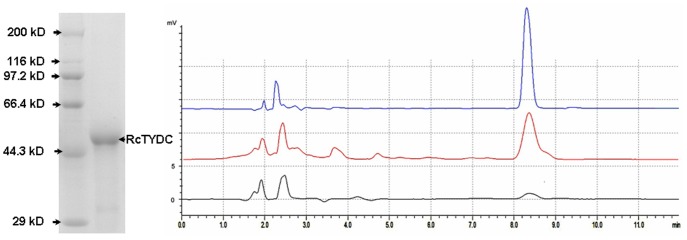
A: recombinant protein of RcTYDC; B: enzymatic assay of recombinant RcTYDC. The blue line was authentic tyramine HPLC trace, the red line was tyramine HPLC trace with feeding tyrosine to recombinant RcTYDC; the grey line was HPLC trace without feeding tyrosine to recombinant RcTYDC.

In order to confirm that the *RcTYDC* gene indeed encoded a functional TYDC protein, the coding region of *RcTYDC* was expressed in *E. coli*. The His_6_-tagged RcTYDC protein was produced under standard conditions using *E. coli* Rosetta cells transformed with the bacterial expression vector pET28 carrying the RcTYDC cDNA. SDS-PAGE revealed an IPTG inducible band at around 53 kDa (identical with the calculated molecular weight of RcTYDC) from 1 h to 6 h, and optimal yield of recombinant protein was obtained at 6 h (Figure 5A). The recombinant RcTYDC was purified and eluted with 250 mM imidazole by a column of Ni-NTA Sepharose. Based on spectrophotometric measurement of protein concentration in the eluted fraction, it was calculated that the concentration of purified recombinant RcTYDC protein was 0.164 µg/µL. In order to analyze the enzymatic activity of purified recombinant RcTYDC protein, tyrosine was used as intermediate to feed the recombinant RcTYDC, and it was found that the recombinant RcTYDC protein catalyzed the formation of tyramine, while the extract with empty vector pET28 was unable to catalyze the formation of tyramine. The intermediate feeding assay indicated that RcTYDC cDNA indeed encoded a functional TYDC protein (Figure 5B).

### Establishment of Hairy Root Cultures of *R. crenulata*

**Figure 6:**
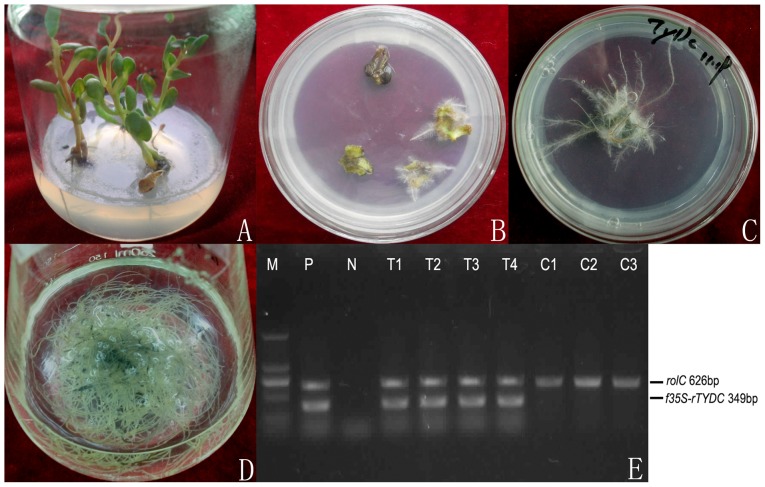
Establishment of transgenic hairy root cultures of *R*. *crenulata* (genomic PCR detection included). A: bacteria-free seedlings; B: induction of hairy roots; C: subcultured hairy root; D: hairy roots cultured in liquid medium; E: genomic PCR analysis of genes of interest.

The transformed roots could emerge from the infected leaves at the wounded sites 10 days after infection with *Agrobacterium* strain C58C1 (Figure 6). The transformed roots, which had lots of lateral branching, could grow very rapidly on hormone-free MS medium (Figure 6), just like previous reports on hairy roots of *Rhodiola sachalinensis* [11]. According to the typical morphologies of hairy root, it might be concluded that the subcultured roots were the hairy roots genetically transformed by Ri plasmid. The transgenic hairy roots could grow well on half-strength MS medium supplemented with 10 mg/L hygromycin, but the nontransgenic one could not grow and finally died. The hairy root cultures were confirmed by genomic PCR and used for liquid culture. The cultured hairy roots in half-strength liquid MS medium grew well (Figure 6).

### The Gene Expressing Profiles and Accumulation Pattern of Salidroside

TYDC is the upstream enzyme of the salidroside biosynthetic pathway and UDPGT is the downstream enzyme, actually the last one of salidroside pathway. Both *TYDC* and *UDPGT* were analyzed at the transcriptional levels in different tissues including roots, stems, leaves and flowers, and in hair root cultures with treatment of elicitors including MeJA, ABA, SA and glucose.

**Figure 7:**
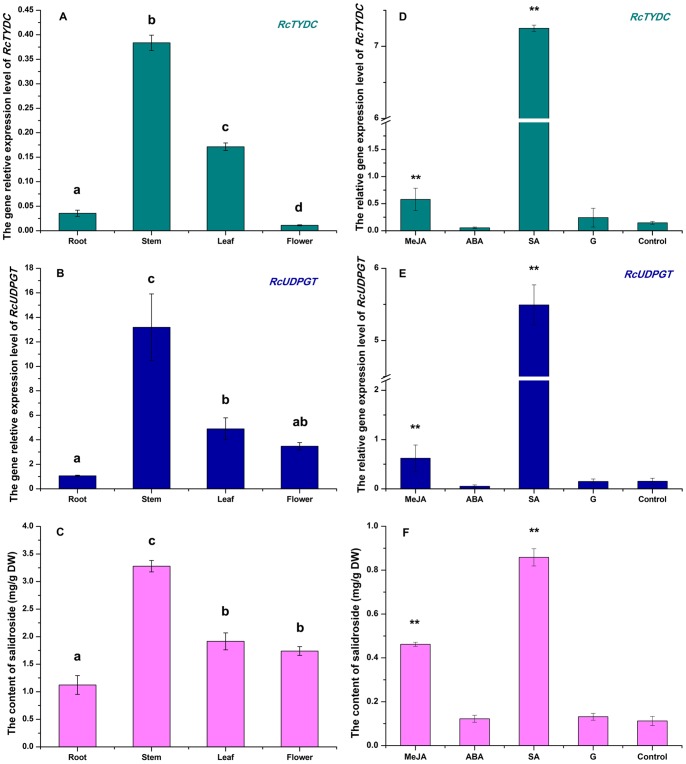
Gene expression levels and salidroside accumulations in different tissues/under different treatments. A: tissue profile of *RcTYDC*; B: tissue profile of *RcUDPGT*; C: the content of salidroside in different tissues of *R*. *crenulata*; D: elicitor-induced profile of *RcTYDC*; E: elicitor-induced profile of *RcUDPGT*; C: the salidroside content of hairy root cultures of *R. crenulata* in different treatments. MeJA: methyl jasmonate; ABA: abscisic acid; SA: salicylic acid; G: glucose. Different letters indicated significant differences (P<0.05) given by Duncan’s multiple range test. ** represented significant difference at P<0.01 given by one sample *t* test. Results represented means±standard deviation (n = 3).

The expression levels of *RcTYDC* and *RcUDPGT* were found in all the four detected tissues including flowers, leaves, stems and roots but at different levels. The tissue expression profile of *RcTYDC* (Figure 7A) was similar with that of *RcUDPGT* (Figure 7B). Both *RcTYDC* and *RcUDPGT* had the highest expression levels in stems. For RcTYDC, the relative expression level in stem was respectively 2, 35 and 11 folds (P<0.01) compared with that in leaf, flower and root (Figure 7A); for RcUDPGT, the relative expression level in stems was respectively 3, 4 and 12 folds (P<0.01) compared with that in leaves, flowers and roots (Figure 7B). The tissue profile showed that *RcTYDC* expression was coordinate with *RcUDPGT* expression in various tissues. HPLC analysis of salidroside showed that stems had the highest content of salidroside (3.28 mg/g DW), which was respectively 1.7, 1.9 and 2.9 folds (P<0.01) of leaf (1.91 mg/g DW), flower (1.74 mg/g DW) and root (1.12 mg/g DW) (Figure 7C). So, the tissue profiles of gene expression were consistent with the tissue accumulation pattern of salidroside.

*RcTYDC* and *RcUDPGT* also showed highly similar expressing pattern of response to elicitors in hairy root cultures. SA dramatically increased the expressing levels of both *RcTYDC* (Figure 7D) and *RcUDPGT* (Figure 7E), which was respectively 49 and 36 folds of control (P<0.01); MeJA remarkably upregulated expression of both *RcTYDC* and *RcUDPGT* (P<0.01), while their effects on up-regulating *RcTYDC*/*RcUDPGT* expression were not as strong as SA. Both ABA and glucose did not significantly affect *RcTYDC*/*RcUDPGT* expression. *RcTYDC* gene expression was induced coordinately with gene expression of *RcUDPGT*, the last key enzyme involved in salidroside biosynthesis. The coordinate expression of genes was often found in natural product biosynthetic pathways such as terpenoid indole alkaloids in *Catharanthus roseus* [12] and nicotine in tobacco [13]. The highest content of salidroside was also found in SA-treated hairy roots, which was 8.58 mg g^−1^ DW, about 7.63 folds of control (1.12 mg g^−1^ DW) (P<0.01); MeJA increased the salidroside accumulation in hairy roots that produced 4.62 mg g^−1^ DW salidroside, about 4.1 folds of control (P<0.01). ABA and glucose did not significantly change the salidroside accumulation in hairy roots of *R*. *crenulata* (Figure 7F).

### Molecular Analysis of Transgenic Hairy Root Cultures of *R.* *crenulata*

In the transgenic hairy root cultures of *R*. *crenulata* with overexpression of *RcTYDC*, a specific 349-bp fragment of *RcTYDC* and 35S promoter could be amplified and at the same time, the 626-bp fragment of *rol*C gene could also be amplified (Figure 6E). Based on the genomic PCR, four hairy root lines were confirmed. However, only the *rol*C gene could be detected in nontransgenic hairy root lines without overexpressing *RcTYDC* (Figure 6E).

**Figure 8:**
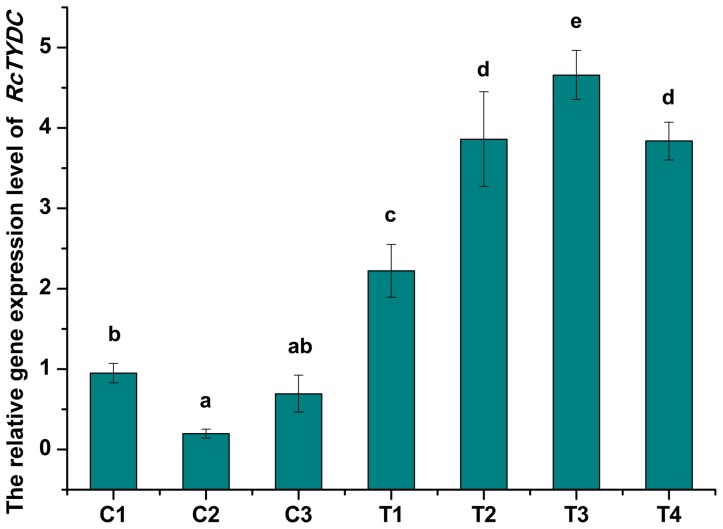
Relative gene expression level of *RcTYDC* in hairy root cultures of *R*. *crenulata*. C: nontransgenic hairy root lines; T: transgenic hairy root lines with overexpressing *RcTYDC*. Different letters indicated significant differences (P<0.05) given by Duncan’s multiple range test. Results represented means±standard deviation (n = 3).

The relative gene expression level was detected in transgenic hairy root lines and non-transgenic lines. The results were shown in Figure 8. Generally, all the transgenic lines with overexpression of *RcTYDC* showed much higher expression level of *RcTYDC* than the non-transgenic ones. The expression levels of *RcTYDC* in transgenic lines were 2.3–21.6 folds compared with that in non-transgenic lines (P<0.01). These results indicated that the cDNA of *RcTYDC* was introduced into *R*. *crenulata* and expressed in corresponding transgenic lines at significantly higher levels that reached the goal of breaking the TYDC-defined step by overexpression and it would facilitate the metabolic flux flowing toward the downstream pathway.

### Detection of Tyramine, Tyrosol and Salidroside by HPLC

**Figure 9:**
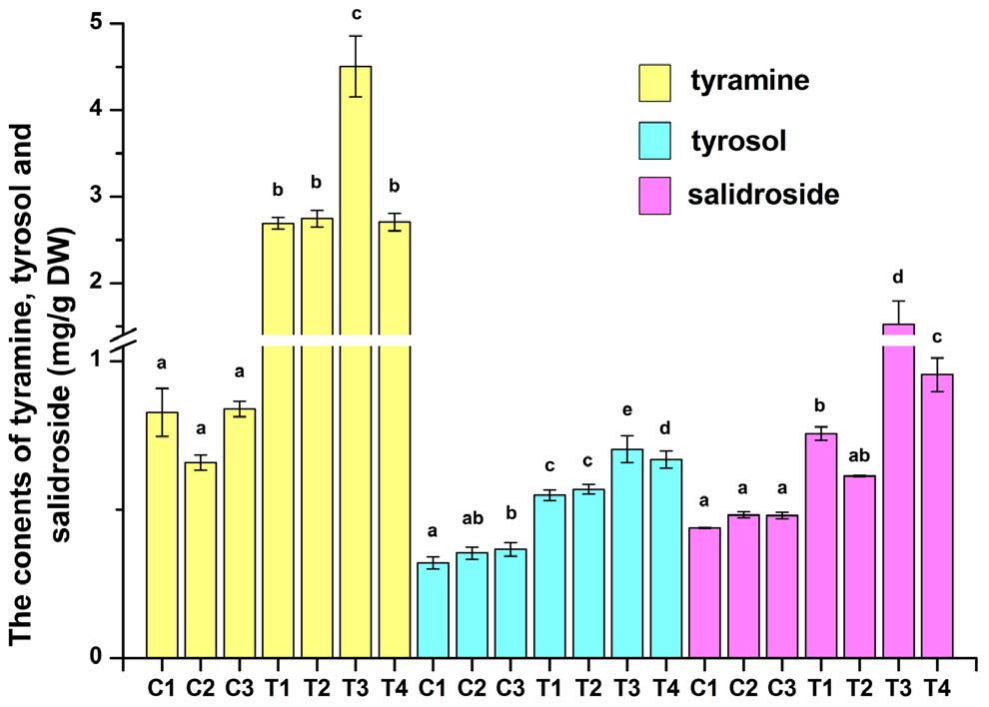
Analysis of metabolites including tyramine, tyrosol and salidroside in hairy root cultures of *R*. *crenulata*. C: nontransgenic hairy root lines; T: transgenic hairy root lines with overexpressing *RcTYDC*. Different letters in tyramine, tyrosol and salidroside contents indicated significant differences (P<0.05) given by Duncan’s multiple range test. Results represented means±standard deviation (n = 3).

To investigate the effects of overexpressing *RcTYDC* on salidroside and its precursor biosynthesis, HPLC was used to analyze the three metabolites including tyramine, tyrosol and salidroside (Figure 9). The non-transgenic hairy root lines produced tyramine from 0.66 mg/g DW to 0.84 mg/g DW; while the transgenic lines yielded tyramine from 2.69 mg/g DW to 4.50 mg/g DW, which was respectively 3.21–6.84 folds (P<0.01) compared with that of non-transgenic lines. This strongly suggested that overexpression of *RcTYDC* could efficiently promote biosynthesis of tyramine and it might facilitate more metabolic flux flowing to the downstream pathway of salidroside biosynthesis. The following HPLC analysis of the downstream intermediate tyrosol and the end-product salidroside proved it. The non-transgenic lines respectively produced tyrosol from 0.32 mg/g DW to 0.37 mg/g DW and salidroside from 0.44 mg/g DW to 0.48 mg/g DW; and transgenic lines respectively produced tyrosol from 0.55 mg/g DW to 0.70 mg/g DW (about 1.50–2.19 folds of non-transgenic lines) and salidroside from 0.61 mg/g DW to 1.52 mg/g DW (about 1.27–3.47 folds of non-transgenic lines). In conclusion, overexpression of *RcTYDC* could remarkably promote biosynthesis of tyramine that facilitated more metabolic flux flowing toward the downstream pathway and as a result, the intermediate tyrosol was accumulated more that led to the increased production of the end-product salidroside.

## Discussion

Metabolic engineering is widely used to produce high-value natural products in a large amount by genetic modification of pathways. Certainly, metabolic engineering is absolutely dependant on gene discovery. Tyrosine decarboxylase catalyzes decarboxylation of tyrosine to generate tyramine as the precursor for salidroside [7]. TYDC operates at an interface between primary and secondary metabolism, suggesting that they possess key regulatory functions in the control of end-product biosynthesis [6]. György *et al* isolated a TYDC gene from *Rhodiola rosea*, of which expression showed consistence with accumulation of salidroside in tissues of different genotype *R*. *rosea* [7]. The *TYDC* gene from *R*. *rosea* was a putative one because only sequence comparison analysis was performed and the authors did not provide the data of enzymatic assay. Unfortunately, it was impossible to confirm that the *TYDC* gene encoded the enzyme of TYDC only at the level of sequence comparison analysis because the sequences of TYDCs and TDCs were similar. The full-length 1670-bp cDNA encoding tyrosine decarboxylase was characterized from Tibet-specific *R*. *crenulata* for the first time. The sequence comparison analysis revealed that RcTYDC was similar with TYDCs of *Opium poppy* and *Citrus* species (about 50% identity) and TDCs of *Catharanthus roseus* (48.2% identity) [14] and *Rauvolfia verticillata* (46.3% identity) [15]. So, the only way to functionally identify TYDC was to feeding intermediate to recombinant TYDC. The recombinant 53 kDa RcTYDC showed the enzymatic activity of TYDC that could convert tyrosine in to tyramine. So, in the present study, a gene encoding TYDC was definitively identified from *R*. *crenulata*.

Metabolic characterization of the genes will be helpful to understand the roles of gene in the pathways. Gene expression analysis is a powerful tool to reveal metabolic characterization of biosynthetic genes. TYDC is the upstream key enzyme and UDPGT, the downstream final-step key enzyme of salidroside biosynthesis [16]. The tissue expression patterns of *TYDC* and *UDPGT* were highly similar: both *TYDC* and *UDPGT* were expressed at much higher levels in stems than in other tissues. The elicitor-induced expression profiles of *TYDC* and *UDPGT* were also highly similar: both *TYDC* and *UDPGT* were dramatically upregulated at the transcriptional level with the treatment of SA and remarkably regulated by MeJA, but not affected by ABA and glucose. So, it could be concluded that *TYDC* expression was coordinate with *UDPGT* expression. The coordinate expression of genes involved in pathway is often found in other plants. The coordinated transcriptional regulation of many terpenoid indole alkaloid synthesizing genes was observed early in different plant organs and in response to positive signals such as elicitors, UV-light and methyl jasmonate in *C*. *roseus* [17]. It was due to the octadecanoid-responsive Catharanthus AP2/ERF-domain (ORCA) transcription factors that regulated the coordinate expression of the TIAs synthesizing genes [12]. In purple-fleshed sweetpotato, the six genes involved in anthocyanin biosynthesis were also coordinately expressed in storage roots controlled by a transcription factor named MYB1 [18]. Obviously, the coordinate expression of genes involved in pathways at higher levels will be helpful to synthesize natural products at higher levels. In the present study, higher expression of both *TYDC* and *UDPGT* led to higher accumulation of salidroside.

The strategy of metabolic engineering by breaking the rate-limiting steps has been successfully using to genetically modify biosynthetic pathways to produce high-valued natural products via overexpressing the rate-limiting enzymes. H6H is the final rate-limiting enzyme involved in scopolamine biosynthesis [19]. Overexpression of *H6H* led to a dramatic increase of scopolamine (about 5-fold higher than scopolamine in non-transgenic plants) in transgenic plants Atropa belladonna [20]. DXS is the first enzyme of the MEP pathway that provides the 5-carcon IPP and DMAPP for diterpenoids [21]. The transgenic hairy root lines with overexpressing *DXS* showed much higher levels of tanshinone production in *Salvia miltiorrhiza*, despite of a very long distance (more than ten enzymatic steps at least) from the DXS-catalyzed step to biosynthesis of the end product tanshinones [21]. So, the strategy of overexpression is successful in metabolic engineering. TYDC is the enzyme at the interface between the primary (amino acid) and secondary (salidroside) metabolism. Generally, the enzymes at the interface between the primary and secondary metabolism possess key regulatory functions in the control of end-product biosynthesis [6]. In the present study, the cDNA of *TYDC* from *R*. *crenulata* was inserted into the genome of hairy root cultures of *R*. *crenulata* with the control of the constitutive CaMV 35S promoter, in order to influence salidroside production. All the transgenic hairy root lines with *RcTYDC* overexpression showed higher expression level of *RcTYDC* than the non-transgenic lines and this suggested that the biosynthetic pathway of salidroside was genetically modified by overexpressing *RcTYDC*. It was reasonable to find that the contents of tyramine (the direct product given by TYDC) were significantly higher in all the *RcTYDC*-transformed hairy root lines than those in non-transgenic lines. This was due to overexpression of *RcTYDC*. HPLC analysis showed that *RcTYDC*-overexpressed hairy root lines also produced tyrosol (the intermediate of salidroside) at a higher level than non-transgenic lines. This suggested that overexpression of *TYDC* directed more metabolic flux to the biosynthetic pathway of salidroside and as a result, the downstream intermediate tyrosol was accumulated more. Finally, overexpression of *RcTYDC* led to an increased accumulation of the end-product salidroside in hairy root cultures of *R*. *crenulata*. The transgenic results suggested that TYDC played a key regulation role in salidroside biosynthesis, which was successfully used to genetically modify the biosynthetic pathway of salidroside and improve the production of salidroside.

## Conclusions

The gene encoding an aromatic amino acid decarboxylase was cloned, metabolically characterized and functionally identified to be tyrosine decarboxylase from Tibet herbal plant species (*Rhodiola crenulata*), which was successfully employed to engineering salidroside biosynthetic pathway of *R*. *crenulata*. The recombinant tyrosine decarboxylase of *R*. *crenulata* converted tyrosine into tyramine, which initialized biosynthesis of salidroside. *RcTYDC* had highest expression level in stems and higher expression level in leaves than that in flowers and roots; and *RcUDPGT* (the last gene involved in salidroside biosynthesis) the highly similar expression patter with that of. The plant growth regulators including SA and MeJA greatly increased expression levels of *RcTYDC* and *RcUDPGT*. These suggested that the coordinate expression of *RcTYDC* and *RcUDPGT*. Furthermore, the gene expressing levels were consistent with the salidroside accumulation levels. *RcTYDC* overexpression promoted tyramine biosynthesis that facilitated more metabolic flux flowing toward the downstream pathway and as a result, the intermediate tyrosol was accumulated more that led to the increased production of the end-product salidroside. So, it was conclude that tyrosine decarboxylase played a key role in salidroside biosynthesis.

## Materials and Methods

### Plant Materials

The plant species of *Rhodiola crenulata* is an endangered plant species in Tibet. Tibet Sciences and Technology Committee approved the research. The mature seeds of *R*. *crenulata* were harvested on the north side of 5000-meter-high Himalaya Mountain in Nyingchi of Tibet in October of 2010. The sterilized seeds germinated and grew into seedlings on MS solid medium at 20°C under 16 h photoperiod and 55 µmol/m^2^/s^1^ light extensity. When the plant seedlings grew to 5–8 cm, the fresh leaves were used for genetic transformation. The flowers, leaves, stems and roots of the mature plants were collected in wild on 5000-meter-high northern side of Himalaya Mountain in Nyingchi of Tibet in July of 2011; and all the tissues were immediately put in liquid nitrogen for storage.

### Gene Cloning

Total RNAs from the whole plant of *R*. *crenulata* were isolated with the RNAplant plus kit (Tiangen, Beijing, China). SMARTTM RACE cDNA Amplification Kit were used to synthesize the single-stranded cDNAs, as templates for PCR amplification of the core cDNA fragment, 3′-RACE and 5′-RACE of the gene of interest, respectively, according to the manufacturer’s protocol (ClonTech, California, USA). Two degenerate primers of dfp (5′-T(G/C)GATG(G/T/A)CCA(A/T/C)GATGC(T/A)CAACCTT-3′) and drp (5′-ACG(A/T)CTCA AC(G/C/A)ATATCT(A/G)GCCAGTC-3′) were used for the standard gradient PCR (from 54 to 62°C) to clone the core cDNA fragment on MyCycler (BIO-RAD, California, USA). Gene specific primers were designed for the rapid amplification of cDNA ends (RACE) based on the core cDNA fragment sequence information. The primer pairs for each 5′-RACE PCR reaction were as follows: forward, the universal primers (UPM and NUP) provided by the kit; reverse, RcTYDC5-1 (5′- GTATTTGCTTGCAGAACGCCACCG-3′) for the first 5′-end amplification, RcTYDC5-2 (5′-GATGGAAGGTTAAGCATCTTGGCC-3′) for the nested amplification. The primer pairs for 3′-RACE PCR were: forward, RcTYDC3-1 (5′-TCTCCTAACGCCCTAATCGAATCC-3′) for the first 3′-end PCR amplification, RcTYDC3-2 (5′- CAAGGACTGGCAGATATCGTTGAG-3′) for the nested amplification; reverse, the universal primers (UPM and NUP) provided by the kit. The first and nested PCR procedures were carried out under the conditions described in the protocol. A pair of primers as follows: fRcTYDC (5′-TTAGCTAGAATCTCACTCTTCCAAC-3′) as the forward primer, rRcTYDC (5′-AATCACTGCTATTGCATGACCTAGCA-3′) as the reverse one, were used to confirm the physical cDNA of *RcTYDC*. Each PCR product was subcloned into the pMD-18T vector and sequenced.

### Bioinformatic Analysis

Comparative and bioinformatics analysis of RcTYDC were carried out online at the websites (http://www.ncbi.nlm.nih.gov and http://www.expasy.org). The nucleotide sequence, deduced amino acid sequence and ORF (open reading frame) encoded by *RcTYDC* were analyzed and the sequence comparison was conducted through a database search using the BLAST program [22]. The multiple alignments of RcTYDC and TYDCs from other species were aligned with CLUSTAL X [23] using default parameters. The phylogenetic tree was constructed by MEGA [24].

### Recombination of RcTYDC and Intermediate Feeding

A pair of primers, frecom (5′-CGGAATTCATGGGCAGCTTGCCTTCTCCTAA-3′) with restriction site *Eco*R I and rrecom (5′-CCAAGCTTTTAAGGCACGATGCTTTGAGCT-3′) with restriction site *Hin*d III, were used to isolate the coding sequence of *RcTYDC* by proof-reading DNA polymerase. After confirmed by sequencing, the coding sequence was inserted into *E*. *coli* expression vector pET28 with *Eco*R I and *Hin*d III. The recombinant expression vector pET28 was introduced into *E*. *coli* strain Rosetta and the transformants could be screened by 25 mg/L kanamycin and 100 mg/L chloramphenicol on solid Luria-Bertani (LB) medium. *E. coli* Rosetta cells harboring plasmid pQE28-*RcTYDC* were cultured at 30°C in LB medium supplemented with kanamycin (25 mg/L) and chloramphenicol (100 mg/L) to an OD600 of 0.3–0.4 and then induced with 1 mM IPTG at 30°C. Bacteria were harvested every hour by centrifugation at 3,000 g and suspended in buffer A (20 mM sodium phosphate buffer, pH 7.4, 10% glycerol, 10 mM imidazole, 5 mM β-mercaptoethanol, 0.5 M sodium chloride, 0.1% Triton X-100). The resulting solution was briefly sonicated (5×5 s) for 30 min and the soluble fraction was recovered after centrifugation at 12,000 g for 15 min. The 6-His-tagged proteins were purified by a metal ion-affinity chromatography. The soluble fraction was applied to a column of Ni-NTA Sepharose (Qiagen, Germany) equilibrated with binding buffer (20 mM sodium phosphate buffer, 0.5 M sodium chloride). Following washing steps with 10 mM imidazole in binding buffer (ten gel volumes) and 100 mM imidazole in binding buffer (six gel volumes), the enzyme was eluted with 250 mM imidazole in binding buffer (three gel volumes). The enzymatic assay was performed according to Lehmann and Pollmann [25]. The 100 µL reaction solution included 13 µg (80 µL) recombinant RcTYDC, 10 µL tyrosine (20 mM), 10 µL pyridoxal phosphate (10 mM), which was immersed in a constant temperature bath for 30 min; and then 10 µL NaOH (2 M) was added to stop the reaction. The 200 µL acetic ether was used for extraction; then the supernatant acetic ether was collected and dried; finally 50 uL methanol was used to dissolve the products. The 10 uL methanol solution was used for HPLC analysis. The control was 80 µL binding buffer without recombinant protein. The HPLC was LC-6AD of Shimadzu and the column was Synergi 4 µ Hydro-RP 80A (250×4.6 mm). The flowing phage was 52% methanol:48% trisodium phosphateis (20 mM) and the flowing rate was 0.8 mL/min. The wavelength of detection was 280 nm.

### Establishment of Hairy Root Cultures of *R. crenulata*

The coding sequence of *RcTYDC* was cloned with a pair of primers (forward primer: 5′- GAAGATCTATGGGCAGCTTGCCTTCTCCTA-3′ and reverse primer: 5′-GAGGTGACCTTAAGGCACGATGCTTTGAG-3′) and inserted into the plant expression vector pCAMBIA1304 with two restriction enzymes, *Bgl* II and *Bst*E II. The constructed plant expression vector was p1304-RcTYDC. Sterile leaf discs of *R*. *crenulata* were inoculated with *A*. *tumefaciens* strain C58C1 (pRiA4 and p1304-RcTYDC). The genetic transformation was performed according to Yang *et al* [4]. Roots generated at cutting edges two to three weeks after co-cultivation were excised and cultured on solid, hormone-free half strength MS medium, supplemented with 30 g/L sucrose as the carbon source. The culture media contained 250 mg/L carbenicillin to eliminate bacteria and 10 mg/L hygromycin as selective pressure for screening the transgenic hairy root lines (integrity of *RcTYDC* into the genome of *R*. *crenulata* confirmed by genomic PCR); and the same media without 10 mg/L hygromycin was used to culture the hairy root lines, of which most were non-transgenic lines (genomic PCR detection of *RcTYDC* were negative). Root culture clones were maintained at 25°C in dark and routinely subcultured every 30 days. The rapidly growing root clones with no bacterial contamination were used to establish transgenic hairy root lines. About 20 mg of fresh roots of about 3 cm in length were inoculated in 250 mL conical flasks containing 50 mL of liquid half-strength MS medium and maintained on an orbital shaker at 100 rpm and 25°C in dark. Four weeks later, the hairy roots cultures were harvested for analysis.

### Treatment of Non-transgenic Hairy Root Cultures With Elicitors

The non-transgenic hairy root cultures from a single clone were used for elicitor treatment. The culturing methods were the same as those described above. Four weeks later, the hairy root cultures were respectively treated with 100 µM MeJA, 50 µM ABA, 20 µM SA and 20 g/L glucose for 24 h. After treatment, the hairy root cultures were respectively harvested for future analysis.

### Detection of the Relative Gene Expression Levels of RcTYDC and RcUDPGT

To investigate the different expression levels of *RcTYDC* and *RcUDPGT* in different tissues including root, stem, leaf and flower, the qPCR was respectively carried out with a pair of primers for *RcTYDC* (forward primer: 5′-TTGACGGAGTTGACCTTGTTG-3′) and reverse primer: 5′- CCAGTCCTTGTAATCCACCATCTC-3′); and the other pair of primers for *RcUDPGT* (forward primer: 5′-CCTGGTGGTGACTTGGTGTAAC-3′ and reverse primer: 5′-GCATTCGTCGGCTGGTCAG-3′). Total cDNAs for each sample were synthesized in a 10 µL reaction mixture using 25 pmol random 9 mers and AMV Reverse Transcriptase (Takara, Japan) at 30°C for 10 min, 42°C for 30 min, 95°C for 5 min and 5°C for 5 min. The qPCR was performed using SYBR Premix ExTaq (Takara, Japan) in a 20 µL final volume containing 5 µL of diluted template cDNA (10×), Premix ExTaq, and the primers (0.2 mM) under the following conditions: initial denaturizing at 94°C for 2 min, 40 cycles of denaturizing at 94°C for 15 s, annealing at 58°C for 15 s and followed by extension at 68°C for 20 s. After each run, a melting curve was analyzed to determine the specificity of amplification by heating the samples from 60 to 95°C. The reaction was carried out on an iQ5 real-time qPCR system (Bio-Rad, USA). All samples were analyzed in three replicates. Data were normalized according to the expression level of *18S rRNA* using 18SF (5′-ATGATAACTCGACGGATCGC-3′) and 18SR (5′-CTTGGATGTGGTAGCCGTTT-3′) primers. The relative gene expression levels of *RcTYDC* and *RcUDPGT* in the treatment of MeJA, ABA, SA and glucose were also performed according to the methods described above. This qPCR analysis was performed at least three replicates.

### Molecular Analysis of Transgenic Hairy Root Lines of *R.* *crenulata*

The genomic PCR was employed to confirm integrity of *rol* genes into the genome of *R*. *crenulata*, which caused the formation of hairy roots. The genomic PCR was performed according to our previously report [26]. At the same time, a pair of primers was used to confirm integrity of *RcTYDC* into hairy roots of *R*. *crenulata*; the primers were: f35s 5′-TTCATTTGGAGAGAACACGGG-3′ as the forward primer that was from the 35S promoter and rTYDC 5′-TTGCTGGAACTGGCTGG-3′ as the reverse primer from the coding sequence of *RcTYDC*. The relative gene expression level of *RcTYDC* in transgenic hairy root lines or non-transgenic ones were detected according the method described above.

### HPLC Analysis of Tyramine, Tyrosol and Salidroside

The content of tyramine was detected by HPLC method based on Simin-Sarkadi’s method [27]. The harvested hairy root cultures were dried at 60°C and then finely powdered. The ultrasonic extracts of acetic ester were distilled and then dissolved in 5 mL menthol. The methanol solvent was filtered through 0.22 µM film, which could be used for HPLC analysis of tyramine. The flowing phase was methanol:20 mM sodium phosphate (V:V = 52%:48%), in which contained 0.1% SDS and the pH was 6.3. The column temperature was 40°C; the flowing rate was 0.8 mL/min and the detecting wavelength was 280 nm. The contents of tyrosol and salidroside were analyzed according to the previous research [28]. The plant materials were dried at 60°C and then finely powdered. The powdered plant materials were dipped in distilled water for 15 min and then autoclaved at 130°C for 30 min. The autoclaved materials were shaken at 37°C for 1 h at the speed of 200 rpm. The supernatant was collected after centrifuge (4000 rpm) and then filtered through 0.22 µM film, which could be used for analysis of tyrosol and salidroside. The flowing phase was water:methanol (V:V = 70%:30%). The column temperature was 40°C; the flowing rate was 1 mL/min and the detecting wavelength was 235 nm. All the samples of injection were 10 µL in volume. There were at least three replicates in HPLC analysis.

### Statistical Analysis

All the experiments including culture of hairy root lines, PCR identification, qPCR analysis of gene expression, HPLC analysis of metabolite were repeated three times. The relative expression levels of the targeted genes and product contents were presented as mean values ± SD. The statistical significance of gene expression in different organs, salidroside content in different organs, RcTYDC expression levels in control/transgenic lines, tyramine, tyrosol and salidroside content in control/transgenic lines were analyzed by Duncan’s multiple range tests. The statistical significance of gene expression with/without treatments was analyzed by one sample *t* test.
